# A putative antimicrobial peptide from Hymenoptera in the megaplasmid pSCL4 of *Streptomyces clavuligerus* ATCC 27064 reveals a singular case of horizontal gene transfer with potential applications

**DOI:** 10.1002/ece3.4924

**Published:** 2019-01-30

**Authors:** Sebastián Ayala‐Ruano, Daniela Santander‐Gordón, Eduardo Tejera, Yunierkis Perez‐Castillo, Vinicio Armijos-Jaramillo

**Affiliations:** ^1^ Universidad San Francisco de Quito, Colegio de Ciencias Biológicas y Ambientales (COCIBA‐USFQ) Quito Ecuador; ^2^ Carrera de Ingeniería en Biotecnología, Facultad de Ingeniería y Ciencias Aplicadas Universidad de Las Américas Quito Ecuador; ^3^ Grupo de Bio‐Quimioinformática Universidad de Las Américas Quito Ecuador; ^4^ Ciencias Físicas y Matemáticas‐Facultad de Formación General Universidad de Las Américas Quito Ecuador

**Keywords:** antimicrobial peptide, horizontal gene transfer, Hymenoptera, pSCL4, *Streptomyces clavuligerus* ATCC 27064

## Abstract

*Streptomyces clavuligerus* is a Gram‐positive bacterium that is a high producer of secondary metabolites with industrial applications. The production of antibiotics such as clavulanic acid or cephamycin has been extensively studied in this species; nevertheless, other aspects, such as evolution or ecology, have received less attention. Furthermore, genes that arise from ancient events of lateral transfer have been demonstrated to be implicated in important functions of host species. This approximation discovered relevant genes that genomic analyses overlooked. Thus, we studied the impact of horizontal gene transfer in the *S. clavuligerus* genome. To perform this task, we applied whole‐genome analysis to identify a laterally transferred sequence from different domains. The most relevant result was a putative antimicrobial peptide (AMP) with a clear origin in the Hymenoptera order of insects. Next, we determined that two copies of these genes were present in the megaplasmid pSCL4 but absent in the *S. clavuligerus* ATCC 27064 chromosome. Additionally, we found that these sequences were exclusive to the ATCC 27064 strain (and so were not present in any other bacteria) and we also verified the expression of the genes using RNAseq data. Next, we used several AMP predictors to validate the original annotation extracted from Hymenoptera sequences and explored the possibility that these proteins had post‐translational modifications using peptidase cleavage prediction. We suggest that Hymenoptera AMP‐like proteins of *S. clavuligerus* ATCC 27064 may be useful for both species adaptation and as an antimicrobial molecule with industrial applications.

## 1 INTRODUCTION

*Streptomyces* is a genus of Gram‐positive bacteria characterized by its ability to produce antitumorals, antihypertensives, immunosuppressives and, above all, antibiotics (Procópio, da Silva, Martins, de Azevedo, & de Araújo, 2012). *Streptomyces clavuligerus* is one of the most important species within the genus for its industrial and clinical applications. This microorganism was isolated from South American soil samples and first described by Higgens and Kastner (1971). In addition to other members of the *Streptomyces* genus, it exhibits a very complex life cycle that includes multicellular, spore‐bearing hyphae and semi‐dormant spores that can survive for long periods in the soil (Bobek, Šmídová, & Čihák, 2017). Moreover, it was described that *S. clavuligerus* produces more than 20 secondary metabolites, including cephamycin C, holomycin, deacetoxycephalosporin C, penicillin N, and tunicamycin, among others (Table 1).

**Table 1:** Antibiotics produced by *Streptomyces clavuligerus*

	Antibiotic	Reference
β‐lactam	Cephamycin C	Nabais and da Fonseca (1995)
β‐lactam	Penicillin N	Nabais and da Fonseca (1995)
β‐lactam	Clavulanic acid	Reading and Cole (1977)
β‐lactam	Cephalosporin	Aharonowitz and Demain (1978)
β‐lactam	Deacetoxycephalosporin C	Nabais and da Fonseca (1995)
β‐lactam	Ro 22‐5417	Pruess and Kellett (1983)
β‐lactam	O‐carbamoyl‐deacetylcephalosporin C	Nabais and da Fonseca (1995)
Non β‐lactam	Holomycin	Li and Walsh (2010)
Non β‐lactam	MM 19290 (related to tunicamycin)	Kenig and Reading (1979)

*Streptomyces clavuligerus* ATCC 27064 contains a linear chromosome (6.7 Mb), three linear short plasmids, pSCL1 (10.5 kb), pSCL2 (149.4 kb), and pSCL3 (442.2 kb), and a linear megaplasmid named pSCL4 (1.8 Mb) (Song et al., 2010). These plasmids can recombine with the chromosome, enhancing the genetic interchange of secondary metabolites (Medema et al., 2010). Interestingly, *S. cattleya* NRRL 3841 and *S. lipmannii* NRRL 3584 are two species of the genus lacking plasmids (Netolitzky, Wu, Jensen, & Roy, 1995), and even the strain *S. clavuligerus* F613‐1 has a different number of plasmids (Cao et al., 2016). These findings suggest that plasmids could provide a vehicle for genetic interchange by horizontal gene transfer or some other mechanism in this species and even in the genus.

Horizontal gene transfer (HGT) corresponds to the transmission of genetic information between related or unrelated organisms (Soucy, Huang, & Gogarten, 2015). This mechanism has been recognized as a driving evolutionary force, and it allows for gene flow between distant evolutionary taxa (Boto, 2010; Syvanen, 2012). Therefore, HGT provides a new combination of sequences that usually confer selective advantages to the host, and it can survive in the “new” genome for long periods of time (Soucy et al., 2015). Furthermore, there are neutral transferred genes that do not confer an immediate benefit but could provide material for variation and posterior innovations (Boto, 2010; Syvanen, 2012).

Horizontal gene transfer has been associated with prokaryotes, so their processes (conjugation, transformation, and transduction) are well understood. However, the advent of sequencing technologies has uncovered a lot of evidence to show that this process also occurs in eukaryotes (Dunning Hotopp et al., 2007; Hirt, Alsmark, & Embley, 2015; Husnik et al., 2013; Sieber, Bromley, & Dunning Hotopp, 2017; Wu et al., 2013). Additionally, there are many instances of HGT events in eukaryotes, for instance the transfer of genes from organelles, such as chloroplasts and mitochondria, to the nucleus (Kleine, Maier, & Leister, 2009) or certain reported cases of gene transmission that occurs in symbiotic relationships between prokaryotes and eukaryotes (Acuña et al., 2012; Boto, 2014; Dunning Hotopp, 2011; Klasson et al., 2014). Examples of HGT involving animals are less detected, presumably because the germline is isolated from somatic cells, meaning that contact with foreign DNA is reduced (Acuña et al., 2012). Despite this observation, several studies have reported animals as acceptors in this process. Genomic sections of the endosymbiotic bacteria *Wolbachia pipientis* have been found in fruit flies and wasps (Conner et al., 2017; Dunning Hotopp et al., 2007). Anecdotally, HGT in the opposite direction was also observed because genomic regions of the mosquito *Aedes aegypti* were detected in *W. pipientis*. Data support the hypothesis that those genes are expressed after an extended evolutionary period because the transfer, with functional significance, produces evolutionary innovation (Woolfit, Iturbe‐Ormaetxe, McGraw, & O'Neill, 2009).

Antimicrobial peptides (AMPs), also known as host defense peptides, are molecules from the innate immune system of all living organisms that protect against a host of bacterial, yeast, fungal, and viral infections and modulate immune responses during infections (Zhang & Gallo, 2016). The best‐known mode of action of these molecules involves the membrane disruption of microbes by forming cavities and producing cell death (Mahlapuu, Håkansson, Ringstad, & Björn, 2016). However, mechanisms affecting intracellular processes such as cell wall formation, DNA, RNA and protein synthesis, and protein folding have also been described (Cudic & Otvos, 2002; Ho, Shah, Chen, & Chen, 2016; Le, Fang, & Sekaran, 2017). Moreover, certain AMPs have been reported as products of an HGT event. These cases include insect drosomycins and nematode cremycins, which are probably acquired from plants (Zhu & Gao, 2014). The Alo‐3 sequence, which is an antifungal peptide found in many plants and in the animal kingdom only in the beetle *Acrocinus longimanus* (Barbault et al., 2003), is the product of an HGT event proposed to explain the presence of this gene in the beetle (Husnik et al., 2013). Finally, a similar case was suggested for thaumatins from nematodes, ticks, and insects, which probably appeared due to HGT from plants (Petre, Major, Rouhier, & Duplessis, 2011).

Antimicrobial peptides exhibit certain properties that give them extraordinary antimicrobial activity and, in some cases, even antitumoral activity. Their shortness in length (usually <100 residues) enables the easy penetration of membranes. In the case of antibacterial peptides, a net positive charge ensures the electrostatic interaction with negatively charged membranes as well as selectivity action against prokaryotes given the neutrality of eukaryote membranes. Another important AMP characteristic is the amphiphilicity (hydrophobic and hydrophilic regions) that facilitates peptide entrance and membrane lysis (Kang, Kim, Seo, & Park, 2017; Mahlapuu et al., 2016; Sun, Xia, Li, Du, & Liang, 2014). Thus, due to the effectiveness of AMPs against microbes, tumoral cells, and their immunomodulatory capacity, these molecules have emerged as a new approach for the treatment of multidrug resistant bacteria, cancer, and other diseases (Mahlapuu et al., 2016).

Antimicrobial peptides have been classified based on their biological activity (antibacterial, antiviral, antifungal, anticancer, and so on), synthesis machinery (gene encoded and nongene encoded), and different properties, such as peptide charge, length, and hydrophobic content, chemical modifications, and others (Wang, 2017). The most extended classification is based on the secondary structure of peptides, which consists of four families (α‐helices, β‐sheets, αβ structures, and non‐αβ structures) determined by the presence or absence of α and β secondary structures in the three‐dimensional structures (Wang, 2017). However, most AMPs lack a 3D structure; thus, a classification has been proposed considering the connection mode of polypeptide chains (Wang, 2015).

Insects are one of the largest producers of AMPs in nature. This is explained by the lack of an adaptive immune system in these animals, meaning that they need to produce a large amount of AMPs from different types as a defense mechanism against pathogens (Bulet & Stöcklin, 2005; Mylonakis, Podsiadlowski, Muhammed, & Vilcinskas, 2016; Yi, Chowdhury, Huang, & Yu, 2014). They release AMPs from the fat body (analogous to a vertebrate's liver) into the hemolymph; thus, they can be distributed throughout the insect body, although other epithelial cells and hemocytes in certain species can also secrete AMPs (Bulet & Stöcklin, 2005). Other important sources of AMPs are insect venom (e.g., melittins and apamins from bees and mastoparans from wasps), which serves as a defense against pathogens or for infecting prey (Moreno & Giralt, 2015), and saliva (e.g., drosomycin from flies and termicin from termites) (Bulet & Stöcklin, 2005; Mylonakis et al., 2016), which protects eggs from infections (Józefiak & Engberg, 2017).

In this study, we report and characterize a putative AMP of the *S. clavuligerus* strain ATCC 27064 transferred from Hymenoptera insects. We used several programs to predict the AMP annotation. Additionally, public RNAseq data were used to corroborate the expression of the transferred gene, and we predicted the potential cleavage sites of the protein in congruence with a post‐translational scenario. To our knowledge, this is the first time that such an event has been reported and provides hints of the complexity of *S. clavuligerus* and its capacity to produce antimicrobial compounds.

## 2 METHODS

### 2.1 Interdomain HGT candidate selection

A BLASTp search was performed (default values, *e*‐value threshold of 1e‐5) using the *S. clavuligerus* strain ATCC 27064 proteome (Song et al., 2010) as the query and UniProt (http://www.uniprot.org) as the database. Next, we retrieved the taxonomy of each blast hit using custom python scripts and a MySQL database. The *S. clavuligerus* proteins with at least 80% of BLAST hits with a taxonomic classification different to bacteria were selected as HGT candidates. The 80% BLAST hit threshold was established through the combination of HGT candidates reported in Richards, Leonard, Soanes, and Talbot (2011) and Schmitt and Lumbsch (2009), allowing for the creation of guidelines to validate the HGT candidates, as proposed in Armijos‐Jaramillo, Sukno, and Thon (2015).

### 2.2 Alignments and phylogenetic reconstruction

We performed global alignment with the BLAST hit candidates obtained in the previous step, using MAFFT 7 (Katoh, Misawa, Kuma, & Miyata, 2002) option auto (offset value 0.123, gap open penalty 1.53, and a BLOSUM62 scoring matrix). Next, we reconstructed a phylogenetic tree using PhyML 3.0 (Guindon et al., 2010) with an LG amino acid replacement matrix, 100 nonparametric bootstrap repetitions, and all other options as default. We manually analyzed the tree topology to establish an HGT pattern in the candidate's trees. DNA alignments were also performed with MAFFT 7 using the same parameters described above.

### 2.3 Contamination elimination and HGT region determination

To avoid obtaining HGT candidates due to contamination in the *S. clavuligerus *genome project, we verified the existence of similar proteins in our HGT candidates through BLASTp searches in the PATRIC database (https://www.patricbrc.org/). The first searches were performed using PATRIC's representative proteome database (*e*‐value threshold 10). Next, we used the *Streptomyces* database and finally the *S. clavuligerus* database, which has available the complete proteome of the two strains (ATCC 27064 from three different projects and F613‐1) plus several plasmids.

To verify the expression of HGT candidates, we used the RNAseq data available in GEO (GSE104738, bioproject PRJNA413703). These data contain deep sequencing of *S. clavuligerus* (strains F613‐1 and ATCC27064) mRNA. We selected the genetic region that encodes one of the HGT protein candidates, and then used this as a template to map readings from RNAseq experiments. To perform the mapping process, we used Geneious mapper (iterations of up to 5 times) from Geneious 10 (http://www.geneious.com; Kearse et al., 2012).

To detect putative HGT regions in the plasmid pSCL4 of *S. clavuligerus* ATCC 27064, we used the software Alien Hunter (Vernikos & Parkhill, 2006), optimizing predicted boundaries with a change‐point detection 2 state 2nd order HMM.

### 2.4 AMP candidate annotation and post‐translational modification prediction

Annotation of our candidates as AMPs was made through several web servers available for this purpose. To estimate the accuracy of the AMP prediction methods, we selected a list of verified AMPs as positive controls and housekeeping genes as negative controls (see Supporting Information Table S1). The list of positive controls was obtained from the Antimicrobial Peptide Database (APD) (http://aps.unmc.edu/AP) using four arthropod peptides of each AMP family with structural information (α‐helices, β‐sheets, αβ structures, and non‐αβ structures) and four without an available structure in the database. We used these data to calculate the sensitivity and specificity of each AMP predictor web server. In total, we evaluated seven AMP predictors: CAMP3 (Waghu, Barai, Gurung, & Idicula‐Thomas, 2016), AMPA (Torrent et al., 2012), ClassAMP (Joseph, Karnik, Nilawe, Jayaraman, & Idicula‐Thomas, 2012), AntiBP (Lata, Sharma, & Raghava, 2007), MLAMP (Lin & Xu, 2016), AMP Scanner Vr.2 (Veltri, Kamath, & Shehu, 2018), and ADP3 (Wang, Li, & Wang, 2016). We used the servers with the highest sensibility and specificity to predict our candidates as AMPs. The signal peptide prediction was performed with SignalP4.1 (Petersen, Brunak, Heijne, & Nielsen, 2011).

We used the web server PROSPER (https://prosper.erc.monash.edu.au/) to predict the post‐translational modifications for our candidates to generate peptides from proteins. This tool predicts the cleavage sites for different proteases detectable in the query sequence. Additionally, to explore the serine protease machinery of *S. clavuligerus*, we predicted and classified these sequences with BLASTp searches against the MERPOPS database (Rawlings, Barrett, & Bateman, 2012).

## 3 RESULTS AND DISCUSSION

### 3.1 Interdomain HGT candidates in *Streptomyces clavuligerus* ATCC 27064

We applied a pipeline adapted from Armijos‐Jaramillo et al. (2015) and Armijos‐Jaramillo, Santander‐Gordón, Soria, Pazmiño‐Betancourth, and Echeverría (2017) to detect interdomain HGT events in the proteome of the *S. clavuligerus* strain ATCC 27064. Because of this search, we found a hypothetical protein (GenBank: EFG03676) highly similar to antimicrobial peptides (AMPs) of arthropods (60% identical to the most similar protein), particularly from the order Hymenoptera and mostly from the suborder Apocrita. We found this protein only in the *S. clavuligerus* strain ATCC 27064, and no other homolog sequences were detected in other *S. clavuligerus* strains or bacterial species.

We found three paralogs to EFG03676 in the *S. clavuligerus* ATCC 27064 proteome: EDY50506, EFG03588, and EDY49959. These sequences are 97.9% identical at the amino acid level, and all were predicted with a signal peptide. From the NCBI databases, we found that the codifying genes of EDY50506 and EDY49959 are located in the bacterial chromosome, whereas EFG03676 and EFG03588 are located in the pSCL4 megaplasmid. Nevertheless, the similarity between sequences led us to analyze the genomic regions that encode the candidate proteins. Thus, we used the DS570654 contig for the EDY50506 codifying region and DS570647 contig for the EDY49959 codifying region, both part of the BioProject PRJNA28551 (https://www.ncbi.nlm.nih.gov/bioproject/?term=PRJNA28551). At the same time, we used the pSCL4 plasmid contig CM000914 to detect codifying regions of EFG03676 and EFG03588 proteins. The sequence alignment demonstrated that the genes of DS570654 and DS570647 originally annotated as part of the *S. clavuligerus* chromosome are identical to two different pSCL4 plasmid regions (contig CM000914, BioProject PRJNA42475, Medema et al., 2010). Thus, we determined that there are only two copies of Hymenoptera AMP‐like genes in the *S. clavuligerus* ATCC 27064 genome, and both are located in the megaplasmid pSCL4. There is no evidence of the presence of these genes in the chromosome contigs of the sequencing projects PRJNA19249 and PRJNA42475. Additionally, the alignment between pSCL4 plasmid contigs CM000914 (BioPoject PRJNA42475) and CM001019 (BioProject PRJNA19249, Song et al., 2010) showed that the regions containing Hymenoptera AMP‐like genes are identical. The current evidence showed that pSCL4 was originated from the excision of a *S. clavuligerus* chromosome section (Álvarez‐Álvarez, Martínez‐Burgo, Rodríguez‐García, & Liras, 2017). Thus, it could be that the original copy of the gene is in the chromosome and is then excised to the plasmid. An alternative scenario is the appearance of one copy in the pSCL4 megaplasmid without trespassing to the chromosome. In any case, the similarity between these two paralogs suggests a recent duplication event.

**Figure 1:**
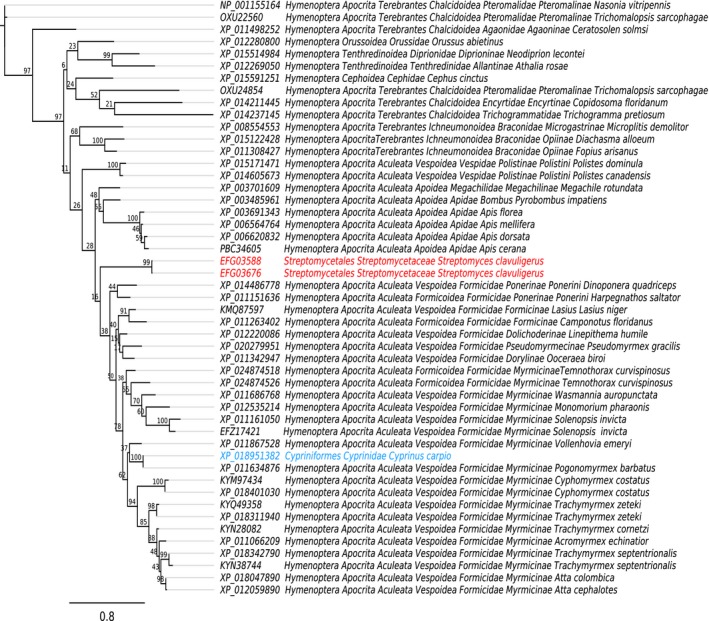
Phylogenetic tree of *Streptomyces clavuligerus* ATCC 27064 Hymenoptera antimicrobial peptide‐like proteins and their homologs. Proteins from bacteria are represented in red, the sequence from Hymenoptera insects are colored in black, and blue highlights the *Cyprinus carpio* (Chordata) sequence*.* IDs of sequence and their taxonomy can be observed in terminal nodes. The values of internal nodes are the nonparametric bootstrap percentage

The presence of Hymenoptera AMP‐like proteins in three different sequencing projects of *S. clavuligerus* ATCC 27064 (BioProjects PRJNA28551, PRJNA42475 and PRJNA19249) demonstrates that those sequences are not artifacts. The lack of EFG03588 homologs in bacteria and their similarity to Hymenoptera AMPs strongly suggests horizontal gene transfer (HGT) as the origin of these molecules in *S. clavuligerus.* The alternative hypothesis (vertical transference) implies the loss of these Hymenoptera AMP‐like genes in all bacterial species except *S. clavuligerus* ATCC 27064, but this explanation is less likely. Additionally, regarding Hymenoptera AMPs, we found a sequence of the common carp *Cyprinus carpio* (XP_018951382) within the homologs of EFG03676‐EFG03588. We tracked XP_018951382 in the scaffold where their codifying gene is located and found no evidence of HGT in the adjacent regions (data not shown). The origin of the arthropod AMP‐like protein of *Cyprinus carpio* is not within the scope of this study, but we suggest that it could be the result of contamination during the sequencing process or a very recent case of HGT. This suggestion is based on the discovery of a 99.2% identity between *C. carpio* and the red harvester ant *Pogonomyrmex barbatus* proteins, and there is no evidence of homology with Chordata species. The phylogenetic relationship of EFG03676 with its paralogs and animal xenologs can be observed in Figure 1. The topology observed in this tree strongly suggests the direction of the transference from Hymenoptera to *S. clavuligerus* or at least this is the last bacterial descendent (discovered so far) that maintains a copy of that gene in its lineage.

Despite the fact that the origin of HGT events cannot be determined with certainty, several reports conclude that close ecological interactions increase the chances of lateral gene transmissions (Degnan, 2014; Venner et al., 2017; Wang & Liu, 2016). In that sense, the interaction between the *Streptomyces* species and Hymenoptera has been constantly reported. That is the case for the fungus‐growing ants, which use antibiotics produced by *Streptomyces* bacteria to protect their cultivations (Currie, Scott, Summerbell, & Malloch, 1999). From there, other protective uses of *Streptomyces* antibiotics have been reported in different Hymenoptera species (de Souza et al., 2013; Engl et al., 2018; Kaltenpoth, Yildirim, Gürbüz, Herzner, & Strohm, 2012; Kroiss et al., 2010; Van Arnam, Currie, & Clardy, 2018). Particularly, *S. clavuligerus* has not been observed in one of these interactions; however, several unidentified *Streptomyces* species were reported by Haeder, Wirth, Herz, and Spiteller (2009) as being associated with leaf‐cutting ants. Some of them were identified as similar to *S. griseus*, a species related to *S. clavuligerus,* which is in agreement with Sembiring (2009). All these arguments lead us to think that a close symbiotic relationship between *S. clavuligerus* and Hymenoptera could lead the transference of AMP‐like gene.

### 3.2 Expression evidence of the HGT candidates

**Figure 2:**
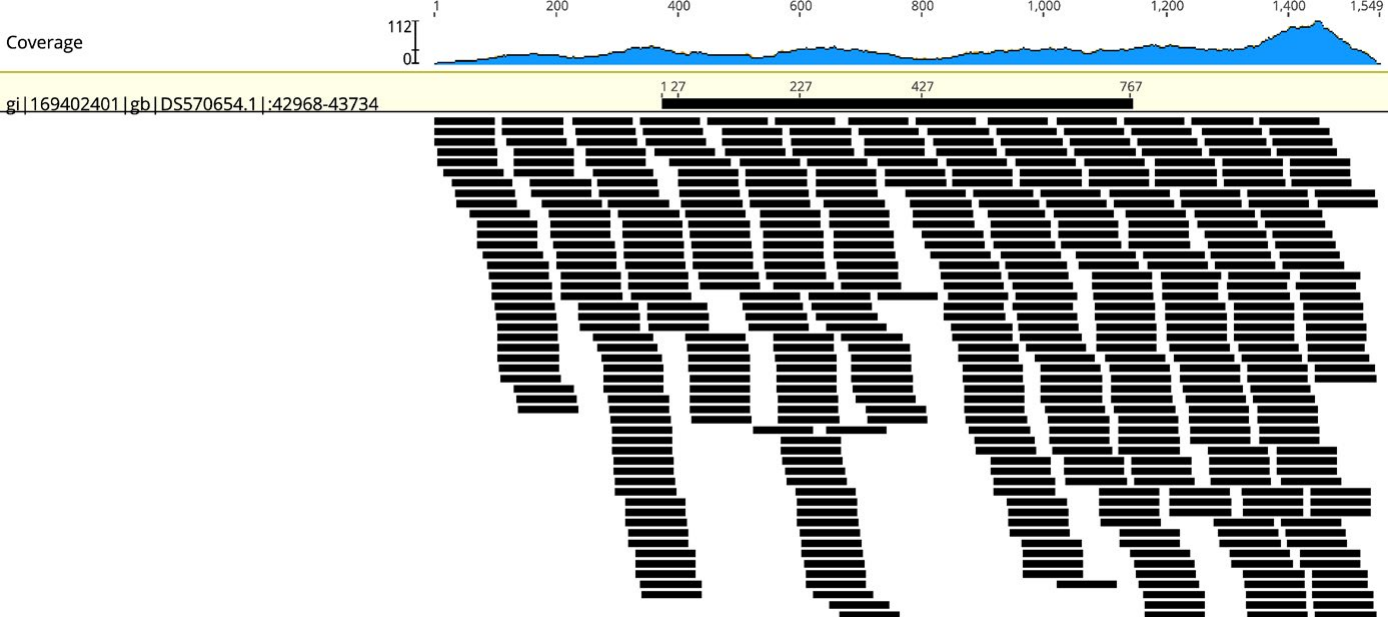
Mapping of the scaffold DS570654 (region from 42,968 to 43,734) of *Streptomyces clavuligerus* ATCC 27064 using RNAseq data (GSE104738). In the upper panel, a graphical representation of the coverage is observed. In the lower panel, the relative position of the mapping readings to the scaffold region is observed

We mapped RNAseq data from *S. clavuligerus* ATCC 27064 to the genomic scaffold DS570654 (BioProject PRJNA28551), from 42,968 to 43,734. This region is the same in the three sequencing projects (PRJNA28551, PRJNA42475, and PRJNA19249) and encodes one of the two copies of Hymenoptera AMP‐like proteins (EFG03588). We observed a clear expression signal along this region (Figure 2). Using RNAseq data from *S. clavuligerus *F613‐1, we did not map readings in the region between 42,968 and 43,734. This result is congruent with the absence of EFG03588 orthologs in bacteria, including other *S. clavuligerus *strains. Additionally, the RNAseq data demonstrate that the codifying gene of EFG03588 is expressed under *in vitro* culture conditions.

### 3.3 Inference of HGT recentness

Lawrence and Ochman (1997) proposed the idea that foreign genes undergo amelioration in order to adapt their sequence to the host genome. This proposition suggests that laterally transferred genes could be differentiated from indigenous ones by several specific features of the donor and receptor genomes. However, by the same effect of amelioration, only “recently” transferred genes could be identified by this approximation because transferred genes over sufficient time (“old” transferred genes) evolve to imitate the receptor genomic features. Considering this argument, we predicted unusual regions in the pSCL4 plasmid genome (contig CM001019). We performed this task through interpolated variable order motifs, a technique implemented in the software Alien Hunter (Vernikos & Parkhill, 2006). This program located six potentially foreign regions in pSCL4: 1–7,500, 19,165–26,714, 46,125–62,763, 73,381–80,082, 102,991–113,397, and 134,187–144,263 (these positions are relative to CM001019). The codifying gene of EFG03588 is located in the region 108,662–109,105 and that of EFG03676 is located in the region 196,642–197,085. Thus, only the codifying gene of EFG03676 is located inside an Alien Hunter HGT predicted region. EFG03676 and EFG03588 are 98% identical; thus, it is improbable that the difference in the predicted regions was produced by these sequences.

Following the idea of amelioration of foreign genes, the Hymenoptera AMP‐like sequences should have sufficient time to adapt to the pSCL4 genome, but there are several other explanations for this observation. In contrast to the amelioration hypothesis, Medrano‐Soto, Moreno‐Hagelsieb, Vinuesa, Christen, and Collado‐Vides (2004) demonstrated that certain transferred genes require a level of codon usage compatibility between the transferred gene and receptor genome to survive over time. Additionally, certain indigenous regions in the genomes possess distinctive features (GC content or codon bias) that can be confused with HGT regions, such as genes with translational robustness (Drummond, Bloom, Adami, Wilke, & Arnold, 2005). Considering these arguments, the lack of HGT signatures in the codifying regions of AMP‐like proteins (particularly EFG03588) might been produced by an amelioration process, codon usage compatibility between donor and receptor or the existence of genomic regions in pSCL4 with certain features that cover up the real HGT regions.

The phylogenetic distribution of the *S. clavuligerus* AMP‐like homologs (Figure 1) suggests that the transfer should occur before the appearance of Apocrita (Hymenoptera suborder). In agreement with the TimeTree database (Kumar, Stecher, Suleski, & Hedges, 2017), this event occurred between 227 and 254 million years ago. The correlation of this time range with HGT occurrence should be considered with precaution because it is based upon the impossibility of associating *S. clavuligerus* proteins with a particular Apocrita clade. When more Apocrita sequences become available, a more precise determination of the time of lateral transfer can be achieved. Additionally, it is possible to identify the specific Apocrita species that underwent transfer. In this scenario, the estimated time of the transfer could be ostensibly lower.

### 3.4 Size estimation of the HGT region

**Figure 3:**
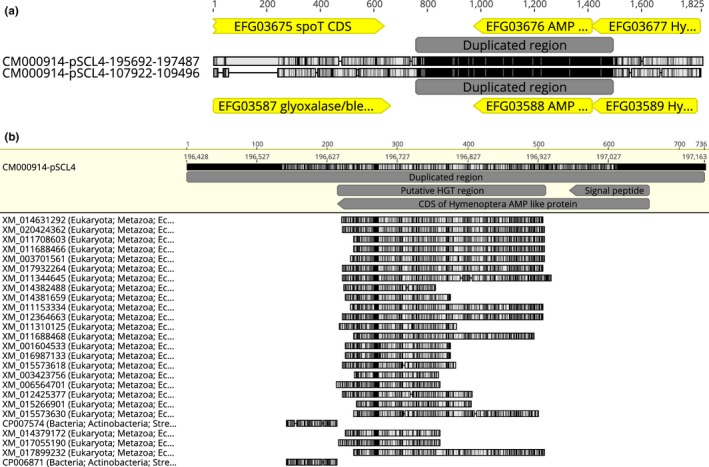
(a) Schematic view of the alignment between CM00914 sections that contain the coding regions of EFG03676–EFG03588 paralogs and their neighbor genes. The section labeled “Duplicated region” shares 97.8% identity. (b) Query centric view of the BLASTn performed on the duplicated region observed in (a). BLAST hits with NCBI codes are shown in the left column with the higher taxonomic level. The region with metazoan hits was annotated as the putative HGT region. The percentage of identity is proportional to the color intensity (with black denoting more likeness and white, less). The genomic annotations are labeled over and under the alignment in yellow or gray

We performed alignment within the codifying sections of Hymenoptera AMP‐like paralogs (EFG03676 and EFG03588) and their neighbor genes in the pSCL4 genome (Figure 3a). In this alignment, we identified a region of 736 bp with 97.8% identity that contains the coding genes of EFG03676 and EFG03588, the last section of the upstream coding genes of the hypothetical proteins EFG03677 and EFG03589 and part of the intergenic section with the downstream genes that codify proteins EFG03675 and EFG03587. Thus, we propose that the entire section of 736 bp was duplicated inside the pSCL4 plasmid.

To explore the size of the HGT event in the current *S. clavuligerus* genome, we performed a BLASTn search using the 736‐bp section annotated as duplicated as a query. We found that only a region of 293 nucleotides has sufficient similarity to retrieve results from Hymenoptera genome sequences (this region excludes the signal peptide) (Figure 3b). In addition, we performed BLASTp searches of the contiguous proteins of AMP‐like sequences (data not shown). The results for EFG03676 and EFG03588 (left contiguous sequences) showed these proteins had a vertical transmission phyletic pattern that was highly similar to that of other *Streptomyces* proteins. By contrast, the BLAST results of EFG03676 and EFG03588 proteins (right contiguous sequences) showed similarity to their own or partial sequences of *S. clavuligerus*. This result shows a misannotation or the possibility that these proteins can be unique to *S. clavuligerus*. In any case, the contiguous sequences to Hymenoptera AMP‐like proteins showed no HGT signal, at least in the current annotation state.

### 3.5 Antimicrobial peptide prediction

To explore whether the HGT candidates maintain the same annotation of their xenologs, we used several AMP prediction software platforms (see Section 2). First, we established the sensibility and specificity of each predictor (Supporting Information Table S1) because we observed heterogeneity in the results obtained from preliminary analysis. Next, we submitted the candidate sequences to the predictors with the highest sensitivity and specificity values (ADP3, AMPA, MLAMP, and AMP Scanner Vr.2).

ADP3 predicted the entire EFG03588 and EFG03676 sequences as AMPs. Its output provides estimations of physicochemical parameters and sequence structure. In the case of HGT candidates, the formation of alpha helices and hydrophobic surfaces was predicted, so they may interact with membranes and function as AMPs. AMPA predicted the peptide TIRFKSQRGHGI (from 126 to 137) of the sequence EFG03676 as an AMP; meanwhile, the sequence EFG03588 was not predicted as an AMP. Both sequences differ in position 134 with G instead of S. Using the regions between 126 and 137 of the sequences EFG03588 and EFG03676, the MLAMP program predicted these peptides as AMPs with a probability of 0.8 and 0.9, respectively, and as antibacterial AMPs with probabilities higher than 0.8 in both cases. AMP Scanner Vr.2 predicted that neither the entire sequence nor the fragments are AMPs.

Despite the increasing number of AMP predictors (Liu, Fan, Sun, Lao, & Zheng, 2017; Porto, Pires, & Franco, 2017) and various approximations used for this task, the diversity of AMPs makes it difficult to use them as an accurate prediction of these types of molecules. Considering this limitation, the prediction of our candidates using 3 of the 4 most sensible programs evaluated in this study makes us confident of the veracity of the annotation.

### 3.6 Post‐translational modification of AMP candidates

Given the predictions of AMPA and MLAMP predictors, we followed the possibility that our candidates were encrypted. These peptides need post‐translational processing from a mature protein in order to be functional (Brand et al., 2012). There is evidence that many AMPs originate from precursor proteins that undergo post‐translational modifications, so these sequences have to undergo cleavage or other modifications in order to be functional (Zhang & Gallo, 2016). Thus, the APD database (http://aps.unmc.edu/AP/) has a collection of 24 different post‐translational modifications that are common among AMPs (Wang et al., 2016). This process has been well characterized in neuroendocrine peptides from vertebrates and invertebrates. These peptides are processed by the cleavage of different types of convertases, such as furin, PC1/3, PC2, PC4, PACE4, and PC5/6 (Veenstra, 2000). These enzymes belong to the family of serine proteases, which have also been implicated in the post‐translational modification of enzymes, hormones, signaling molecules, and growth factors (Małuch, Walewska, Sikorska, & Prahl, 2016).

We performed the prediction of the cleavage sites of our candidate proteins using the web server PROSPER, and we found that they could be cut in different positions by several proteases. Remarkably, the server predicted cutting sites (by serine proteases) near to the AMP prediction of the AMPA and MLAMP programs. Thus, the PROSPER results suggest that an elastase‐2 makes cuts in the sequence EFG03588 at positions 25, 96, 99, 127, 140, and 141 and in EFG03676 at positions 25, 33, 96, 99, 127, 140, and 141. The cleavage of the residues 127 and 141 includes the peptide predicted with AMPA and MLAMP. The resultant peptide of the cleavage in these two positions (RFKSQRG/SHGIDFV) was also predicted as AMP by the AMPA and MLAMP programs.

**Figure 4:**
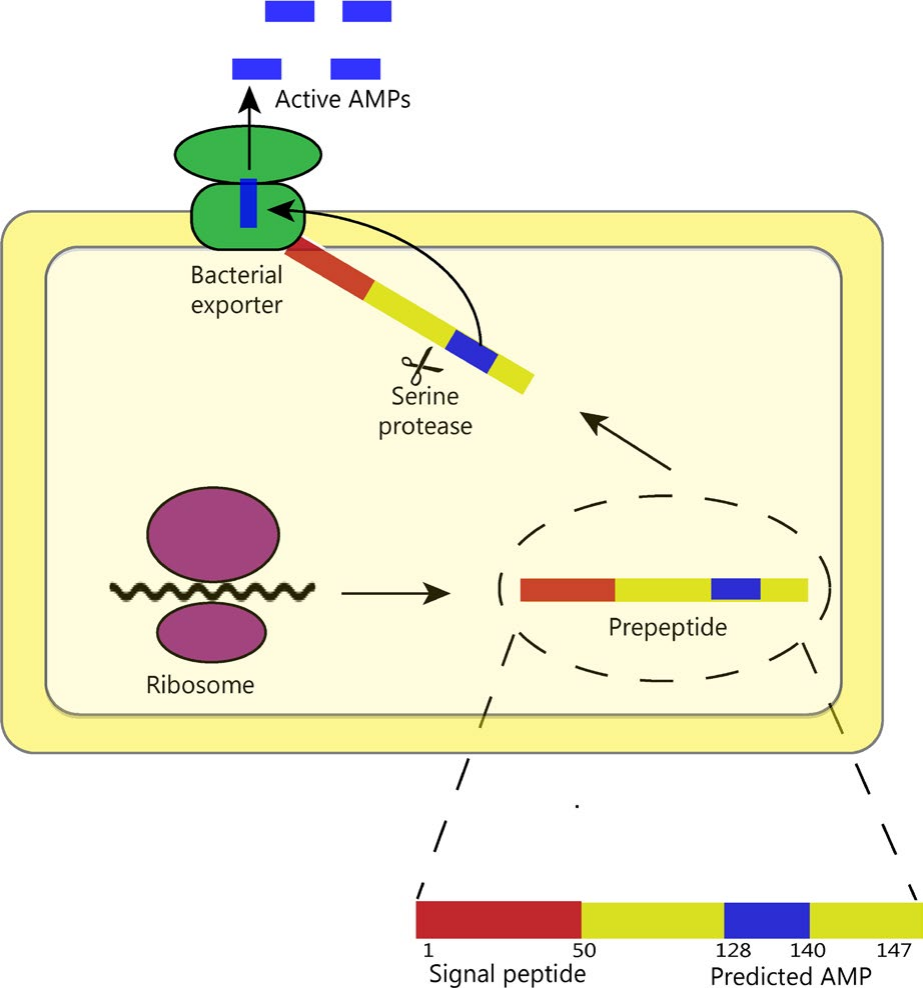
Proposed scenario for the cleavage and secretion of AMPs from EFG03588 and EFG03676 (Hymenoptera AMP‐like proteins)

Elastase‐2 belongs to the serine proteases family, which are widely extended in all kingdoms of life (Tripathi & Sowdhamini, 2008), including *S. clavuligerus*, which has thirteen of these proteins annotated in its proteome. We performed BLASTp searches of *S. clavuligerus *serine proteases in the MEROPS database (https://www.ebi.ac.uk/merops/index.shtml) and found eight serine proteases of the S1 family, four of the S8 family and one of the M6 family. Elastase‐2 belongs to the S1A subfamily, and we found three of these sequences in the *S. clavuligerus* proteome. The presence of these enzymes suggests that *S. clavuligerus* possesses the machinery to cleave EFG03676 and EFG03588 proteins. What is more, the signal peptide of these proteins could be used to translocate the sequence to some transporter (like an ABC transporter) to process the protein and then secrete the peptide outside the cell. A similar mechanism has been observed in class IIa bacteriocins (Drider, Fimland, Héchard, McMullen, & Prévost, 2006; Perez, Zendo, & Sonomoto, 2014). In this scenario, Hymenoptera AMP‐like sequences could be translocated, guided by the signal peptide, and then be submitted to post‐translational proteolytic cleavage using serine proteases (and likely many other enzymes and complexes) before being secreted outside the cell (Figure 4). With this scenario, we highlight the machinery available in bacteria to complete the processing of AMPs even in sequences that arise from distant organisms. In fact, several approaches have been reported to process and secrete polypeptides from longer proteins (Dores, Lecaudé, Bauer, & Danielson, 2002; Fukudome & Yoshikawa, 1992; Ivanov, Karelin, Philippova, Nazimov, & Pletnev, 1997; Lewis & Stern, 1983; Meisel & Bockelmann, 1999; Vanhoye, Bruston, Nicolas, & Amiche, 2003; Zhao, Garreau, Sannier, & Piot, 1997), and only experimental procedures can unveil the real processing of our candidates.

Although some other peptides (especially with cyclic conformation) with antimicrobial and/or antitumoral effects have been reported in the *Streptomyces* genus (Shetty, Buddana, Tatipamula, Naga, & Ahmad, 2014; Um et al., 2013; Zhou et al., 2014), this is the first study to detect an AMP that is laterally transferred in this group of organisms. More than 2,500 natural or encrypted AMPs have been described previously (Zhang & Gallo, 2016). However, some of those have low antimicrobial activity or an unknown function. Therefore, the stable HGT genes were demonstrated to play relevant roles in receptor organisms (Belbahri, Calmin, Mauch, & Andersson, 2008; Friesen et al., 2006; Slot & Rokas, 2010; Tiburcio et al., 2010; Wenzl, Wong, Kwang‐won, & Jefferson, 2005). The rationale behind this observation suggests that the increase in the fitness generated by the HGT gene in the receptor organism compensates or overtakes the negative selection that a gene of this type would undergo, because a foreign gene (especially in the interdomain HGT) should overcome the incompatibility of promoters and an alternative genetic code, without mentioning the cost generated by the synthesis and duplication of new genetic material, especially in prokaryotic genomes that tend to be highly efficient (Kuo & Ochman, 2009; Mira, Ochman, & Moran, 2001). Thus, the presence of Hymenoptera AMP‐like genes in *S. clavuligerus* ATCC 27064 should play an important role in this strain. This assertion is supported by the estimated time in which the Hymenoptera transferred genes are retained in the *S. clavuligerus* genome. Additionally, the presence of two copies in the genome suggests an expansion of the family, perhaps to produce dosage duplication. These lines of evidence lead us to propose that Hymenoptera AMP‐like proteins could be useful antimicrobial molecules for *S. clavuligerus* ATCC 27064 with several biotechnological applications that can be explored.

## CONFLICT OF INTEREST

None declared.

## AUTHOR CONTRIBUTIONS

SA‐R performed experiments and wrote the first draft of the manuscript; DS‐G performed experiments and edited the manuscript; ET and YP‐C helped in experimental design and in manuscript editing; and VA‐J designed and performed the experiments and wrote and edited the manuscript.

## Data Availability

Table S1 is available in the supporting information section.

## References

[ece34924-bib-0001] Acuña, R. , Padilla, B. E. , Flórez‐Ramos, C. P. , Rubio, J. D. , Herrera, J. C. , Benavides, P. , … Rose, J. K. C. (2012). Adaptive horizontal transfer of a bacterial gene to an invasive insect pest of coffee. Proceedings of the National Academy of Sciences USA, 109, 4197–4202. 10.1073/pnas.1121190109 PMC3306691 22371593

[ece34924-bib-0002] Aharonowitz, Y. , & Demain, A. L. (1978). Carbon catabolite regulation of cephalosporin production in *Streptomyces clavuligerus* . Antimicrobial Agents and Chemotherapy, 14, 159–164. 10.1128/AAC.14.2.159 697343 PMC352426

[ece34924-bib-0003] Álvarez‐Álvarez, R. , Martínez‐Burgo, Y. , Rodríguez‐García, A. , & Liras, P. (2017). Discovering the potential of S. clavuligerus for bioactive compound production: Cross‐talk between the chromosome and the pSCL4 megaplasmid. BMC Genomics, 18, 907 10.1186/s12864-017-4289-y 29178826 PMC5702194

[ece34924-bib-0004] Armijos‐Jaramillo, V. , Santander‐Gordón, D. , Soria, R. , Pazmiño‐Betancourth, M. , & Echeverría, M. C. (2017). A whole genome analysis reveals the presence of a plant PR1 sequence in the potato pathogen *Streptomyces scabies* and other *Streptomyces* species. Molecular Phylogenetics and Evolution, 114, 346–352. 10.1016/j.ympev.2016.08.006 27530704

[ece34924-bib-0005] Armijos‐Jaramillo, V. D. , Sukno, S. A. , & Thon, M. R. (2015). Identification of horizontally transferred genes in the genus *Colletotrichum* reveals a steady tempo of bacterial to fungal gene transfer. BMC Genomics, 16, 2 10.1186/1471-2164-16-2 25555398 PMC4320630

[ece34924-bib-0006] Barbault, F. , Landon, C. , Guenneugues, M. , Meyer, J.‐P. , Schott, V. , Dimarcq, J.‐L. , & Vovelle, F. (2003). Solution structure of Alo‐3: A new knottin‐type antifungal peptide from the insect *Acrocinus longimanus* . Biochemistry, 42, 14434–14442. 10.1021/bi035400o 14661954

[ece34924-bib-0007] Belbahri, L. , Calmin, G. , Mauch, F. , & Andersson, J. O. (2008). Evolution of the cutinase gene family: Evidence for lateral gene transfer of a candidate Phytophthora virulence factor. Gene, 408, 1–8. 10.1016/j.gene.2007.10.019 18024004

[ece34924-bib-0008] Bobek, J. , Šmídová, K. , & Čihák, M. (2017). A waking review: Old and novel insights into the spore germination in streptomyces. Frontiers in Microbiology, 8, 2205 10.3389/fmicb.2017.02205 29180988 PMC5693915

[ece34924-bib-0009] Boto, L. (2010). Horizontal gene transfer in evolution: Facts and challenges. Proceedings of the Royal Society B: Biological Sciences, 277, 819–827. 10.1098/rspb.2009.1679 PMC2842723 19864285

[ece34924-bib-0010] Boto, L. (2014). Horizontal gene transfer in the acquisition of novel traits by metazoans. Proceedings of the Royal Society B: Biological Sciences, 281, 20132450 10.1098/rspb.2013.2450 PMC3896011 24403327

[ece34924-bib-0011] Brand, G. D. , Magalhães, M. T. Q. , Tinoco, M. L. P. , Aragão, F. J. L. , Nicoli, J. , Kelly, S. M. , … Bloch, C. (2012). Probing protein sequences as sources for encrypted antimicrobial peptides. PLoS ONE, 7, e45848 10.1371/journal.pone.0045848 23029273 PMC3461044

[ece34924-bib-0012] Bulet, P. , & Stöcklin, R. (2005). Insect antimicrobial peptides: Structures, properties and gene regulation. Protein and Peptide Letters, 12, 3–11. 15638797 10.2174/0929866053406011

[ece34924-bib-0013] Cao, G. , Zhong, C. , Zong, G. , Fu, J. , Liu, Z. , Zhang, G. , & Qin, R. (2016). Complete genome sequence of *Streptomyces clavuligerus* F613–1, an industrial producer of clavulanic acid. Genome Announcements, 4, e01020–16. 10.1128/genomeA.01020-16 27660792 PMC5034143

[ece34924-bib-0014] Conner, W. R. , Blaxter, M. L. , Anfora, G. , Ometto, L. , Rota‐Stabelli, O. , & Turelli, M. (2017). Genome comparisons indicate recent transfer of wRi‐like Wolbachia between sister species *Drosophila suzukii* and *D. subpulchrella* . Ecology and Evolution, 7, 9391–9404. 10.1002/ece3.3449 29187976 PMC5696437

[ece34924-bib-0015] Cudic, M. , & Otvos, L. (2002). Intracellular targets of antibacterial peptides. Current Drug Targets, 3, 101–106. 11958294 10.2174/1389450024605445

[ece34924-bib-0016] Currie, C. R. , Scott, J. A. , Summerbell, R. C. , & Malloch, D. (1999). Fungus‐growing ants use antibiotic‐producing bacteria to control garden parasites. Nature, 398, 701–704. 10.1038/19519

[ece34924-bib-0017] de Lima Procópio, R. E. , da Silva, I. R. , Martins, M. K. , de Azevedo, J. L. , & de Araújo, J. M. (2012). Antibiotics produced by *Streptomyces* . Brazilian Journal of Infectious Diseases, 16, 466–471. 10.1016/j.bjid.2012.08.014 22975171

[ece34924-bib-0018] de Souza, D. J. , Lenoir, A. , Kasuya, M. C. M. , Ribeiro, M. M. R. , Devers, S. , Couceiro, J. C. , … Della Lucia, T. M. C. (2013). Ectosymbionts and immunity in the leaf‐cutting ant *Acromyrmex subterraneus* subterraneus. Brain, Behavior, and Immunity, 28, 182–187. 10.1016/j.bbi.2012.11.014 23207105

[ece34924-bib-0019] Degnan, S. M. (2014). Think laterally: Horizontal gene transfer from symbiotic microbes may extend the phenotype of marine sessile hosts. Frontiers in Microbiology, 5, 638 10.3389/fmicb.2014.00638 25477875 PMC4237138

[ece34924-bib-0020] Dores, R. M. , Lecaudé, S. , Bauer, D. , & Danielson, P. B. (2002). Analyzing the evolution of the opioid/orphanin gene family. Mass Spectrometry Reviews, 21, 220–243. 10.1002/mas.10029 12533798

[ece34924-bib-0021] Drider, D. , Fimland, G. , Héchard, Y. , McMullen, L. M. , & Prévost, H. (2006). The continuing story of class IIa bacteriocins. Microbiology and Molecular Biology Reviews, 70, 564–582. 10.1128/MMBR.00016-05 16760314 PMC1489543

[ece34924-bib-0022] Drummond, D. A. , Bloom, J. D. , Adami, C. , Wilke, C. O. , & Arnold, F. H. (2005). Why highly expressed proteins evolve slowly. Proceedings of the National Academy of Sciences USA, 102, 14338–14343. 10.1073/pnas.0504070102 PMC1242296 16176987

[ece34924-bib-0023] Dunning Hotopp, J. C. (2011). Horizontal gene transfer between bacteria and animals. Trends in Genetics, 27, 157–163. 10.1016/j.tig.2011.01.005 21334091 PMC3068243

[ece34924-bib-0024] Dunning Hotopp, J. C. , Clark, M. E. , Oliveira, D. C. S. G. , Foster, J. M. , Fischer, P. , Muñoz Torres, M. C. , … Werren, J. H. (2007). Widespread lateral gene transfer from intracellular bacteria to multicellular eukaryotes. Science, 317, 1753–1756. 10.1126/science.1142490 17761848

[ece34924-bib-0025] Engl, T. , Kroiss, J. , Kai, M. , Nechitaylo, T. Y. , Svatoš, A. , & Kaltenpoth, M. (2018). Evolutionary stability of antibiotic protection in a defensive symbiosis. Proceedings of the National Academy of Sciences USA, 115, E2020–E2029. 10.1073/pnas.1719797115 PMC5834716 29444867

[ece34924-bib-0026] Friesen, T. L. , Stukenbrock, E. H. , Liu, Z. , Meinhardt, S. , Ling, H. , Faris, J. D. , … Oliver, R. P. (2006). Emergence of a new disease as a result of interspecific virulence gene transfer. Nature Genetics, 38, 953–956. 10.1038/ng1839 16832356

[ece34924-bib-0027] Fukudome, S. , & Yoshikawa, M. (1992). Opioid peptides derived from wheat gluten: Their isolation and characterization. FEBS Letters, 296, 107–111. 1309704 10.1016/0014-5793(92)80414-c

[ece34924-bib-0028] Guindon, S. , Dufayard, J.‐F. , Lefort, V. , Anisimova, M. , Hordijk, W. , & Gascuel, O. (2010). New algorithms and methods to estimate maximum‐likelihood phylogenies: Assessing the performance of PhyML 3.0. Systematic Biology, 59, 307–321. 10.1093/sysbio/syq010 20525638

[ece34924-bib-0029] Haeder, S. , Wirth, R. , Herz, H. , & Spiteller, D. (2009). Candicidin‐producing Streptomyces support leaf‐cutting ants to protect their fungus garden against the pathogenic fungus *Escovopsis* . Proceedings of the National Academy of Sciences USA, 106, 4742–4746. 10.1073/pnas.0812082106 PMC2660719 19270078

[ece34924-bib-0030] Higgens, C. E. , & Kastner, R. E. (1971). *Streptomyces clavuligerus* sp. nov., a β‐lactam antibiotic producer. International Journal of Systematic and Evolutionary Microbiology, 21, 326–331. 10.1099/00207713-21-4-326

[ece34924-bib-0031] Hirt, R. P. , Alsmark, C. , & Embley, T. M. (2015). Lateral gene transfers and the origins of the eukaryote proteome: A view from microbial parasites. Current Opinion in Microbiology, 23, 155–162. 10.1016/j.mib.2014.11.018 25483352 PMC4728198

[ece34924-bib-0032] Ho, Y.‐H. , Shah, P. , Chen, Y.‐W. , & Chen, C.‐S. (2016). Systematic analysis of intracellular‐targeting antimicrobial peptides, bactenecin 7, hybrid of pleurocidin and dermaseptin, proline‐arginine‐rich peptide, and lactoferricin B, by using *Escherichia coli* proteome microarrays. Molecular & Cellular Proteomics, 15, 1837–1847. 10.1074/mcp.M115.054999 26902206 PMC5083092

[ece34924-bib-0033] Husnik, F. , Nikoh, N. , Koga, R. , Ross, L. , Duncan, R. P. , Fujie, M. , … McCutcheon, J. P. (2013). Horizontal gene transfer from diverse bacteria to an insect genome enables a tripartite nested mealybug symbiosis. Cell, 153, 1567–1578. 10.1016/j.cell.2013.05.040 23791183

[ece34924-bib-0034] Ivanov, V. T. , Karelin, A. A. , Philippova, M. M. , Nazimov, I. V. , & Pletnev, V. Z. (1997). Hemoglobin as a source of endogenous bioactive peptides: The concept of tissue‐specific peptide pool. Biopolymers, 43, 171–188. 10.1002/(SICI)1097-0282(1997)43:2<171:AID-BIP10>3.0.CO;2-O 9216253

[ece34924-bib-0035] Joseph, S. , Karnik, S. , Nilawe, P. , Jayaraman, V. K. , & Idicula‐Thomas, S. (2012). ClassAMP: A prediction tool for classification of antimicrobial peptides. IEEE/ACM Transactions on Computational Biology and Bioinformatics, 9, 1535–1538. 10.1109/TCBB.2012.89 22732690

[ece34924-bib-0036] Józefiak, A. , & Engberg, R. M. (2017). Insect proteins as a potential source of antimicrobial peptides in livestock production. A review. Journal of Animal and Feed Sciences, 26, 87–99. 10.22358/jafs/69998/2017

[ece34924-bib-0037] Kaltenpoth, M. , Yildirim, E. , Gürbüz, M. F. , Herzner, G. , & Strohm, E. (2012). Refining the roots of the beewolf‐*Streptomyces* symbiosis: Antennal symbionts in the rare genus *Philanthinus* (Hymenoptera, Crabronidae). Applied and Environment Microbiology, 78, 822–827. 10.1128/AEM.06809-11 PMC3264120 22113914

[ece34924-bib-0038] Kang, H.‐K. , Kim, C. , Seo, C. H. , & Park, Y. (2017). The therapeutic applications of antimicrobial peptides (AMPs): A patent review. Journal of Microbiology, 55, 1–12. 10.1007/s12275-017-6452-1 28035594

[ece34924-bib-0039] Katoh, K. , Misawa, K. , Kuma, K. , & Miyata, T. (2002). MAFFT: A novel method for rapid multiple sequence alignment based on fast Fourier transform. Nucleic Acids Research, 30, 3059–3066. 12136088 10.1093/nar/gkf436 PMC135756

[ece34924-bib-0040] Kearse, M. , Moir, R. , Wilson, A. , Stones‐Havas, S. , Cheung, M. , Sturrock, S. , … Drummond, A. (2012). Geneious basic: An integrated and extendable desktop software platform for the organization and analysis of sequence data. Bioinformatics, 28, 1647–1649. 10.1093/bioinformatics/bts199 22543367 PMC3371832

[ece34924-bib-0041] Kenig, M. , & Reading, C. (1979). Holomycln and an antibiotic (MM 19290) related to tunicamycln, metabolites of *Streptomyces clavuligerus* . Journal of Antibiotics, 32, 549–554. 10.7164/antibiotics.32.549 468729

[ece34924-bib-0042] Klasson, L. , Kumar, N. , Bromley, R. , Sieber, K. , Flowers, M. , Ott, S. H. , … Dunning Hotopp, J. C. (2014). Extensive duplication of the Wolbachia DNA in chromosome four of *Drosophila ananassae* . BMC Genomics, 15, 1097 10.1186/1471-2164-15-1097 25496002 PMC4299567

[ece34924-bib-0043] Kleine, T. , Maier, U. G. , & Leister, D. (2009). DNA transfer from organelles to the nucleus: The idiosyncratic genetics of endosymbiosis. Annual Review of Plant Biology, 60, 115–138. 10.1146/annurev.arplant.043008.092119 19014347

[ece34924-bib-0044] Kroiss, J. , Kaltenpoth, M. , Schneider, B. , Schwinger, M.‐G. , Hertweck, C. , Maddula, R. K. , … Svatos, A. (2010). Symbiotic *Streptomycetes* provide antibiotic combination prophylaxis for wasp offspring. Nature Chemical Biology, 6, 261–263. 10.1038/nchembio.331 20190763

[ece34924-bib-0045] Kumar, S. , Stecher, G. , Suleski, M. , & Hedges, S. B. (2017). TimeTree: A resource for timelines, timetrees, and divergence times. Molecular Biology and Evolution, 34, 1812–1819. 10.1093/molbev/msx116 28387841

[ece34924-bib-0046] Kuo, C.‐H. , & Ochman, H. (2009). Deletional bias across the three domains of life. Genome Biology and Evolution, 1, 145–152. 10.1093/gbe/evp016 20333185 PMC2817411

[ece34924-bib-0047] Lata, S. , Sharma, B. , & Raghava, G. (2007). Analysis and prediction of antibacterial peptides. BMC Bioinformatics, 8, 263 10.1186/1471-2105-8-263 17645800 PMC2041956

[ece34924-bib-0048] Lawrence, J. G. , & Ochman, H. (1997). Amelioration of bacterial genomes: Rates of change and exchange. Journal of Molecular Evolution, 44, 383–397. 9089078 10.1007/pl00006158

[ece34924-bib-0049] Le, C.‐F. , Fang, C.‐M. , & Sekaran, S. D. (2017). Intracellular targeting mechanisms by antimicrobial peptides. Antimicrobial Agents and Chemotherapy, 61, e02340–16. 10.1128/AAC.02340-16 28167546 PMC5365711

[ece34924-bib-0050] Lewis, R. V. , & Stern, A. S. (1983). Biosynthesis of the enkephalins and enkephalin‐containing polypeptides. Annual Review of Pharmacology and Toxicology, 23, 353–372. 10.1146/annurev.pa.23.040183.002033 6307125

[ece34924-bib-0051] Li, B. , & Walsh, C. T. (2010). Identification of the gene cluster for the dithiolopyrrolone antibiotic holomycin in *Streptomyces clavuligerus* . Proceedings of the National Academy of Sciences USA, 107, 19731–19735. 10.1073/pnas.1014140107 PMC2993409 21041678

[ece34924-bib-0052] Lin, W. , & Xu, D. (2016). Imbalanced multi‐label learning for identifying antimicrobial peptides and their functional types. Bioinformatics, 32, 3745–3752. 10.1093/bioinformatics/btw560 27565585 PMC5167070

[ece34924-bib-0053] Liu, S. , Fan, L. , Sun, J. , Lao, X. , & Zheng, H. (2017). Computational resources and tools for antimicrobial peptides. Journal of Peptide Science, 23, 4–12. 10.1002/psc.2947 27966278

[ece34924-bib-0054] Mahlapuu, M. , Håkansson, J. , Ringstad, L. , & Björn, C. (2016). Antimicrobial peptides: An emerging category of therapeutic agents. Frontiers in Cellular and Infection Microbiology, 6, 194 10.3389/fcimb.2016.00194 28083516 PMC5186781

[ece34924-bib-0055] Małuch, I. , Walewska, A. , Sikorska, E. , & Prahl, A. (2016). Proprotein convertases – Family of serine proteases with a broad spectrum of physiological functions. Postepy Biochemii, 62, 472–481. 28132449

[ece34924-bib-0056] Medema, M. H. , Trefzer, A. , Kovalchuk, A. , van den Berg, M. , Müller, U. , Heijne, W. , … Takano, E. (2010). The sequence of a 1.8‐mb bacterial linear plasmid reveals a rich evolutionary reservoir of secondary metabolic pathways. Genome Biology and Evolution, 2, 212–224. 10.1093/gbe/evq013 20624727 PMC2997539

[ece34924-bib-0057] Medrano‐Soto, A. , Moreno‐Hagelsieb, G. , Vinuesa, P. , Christen, J. A. , & Collado‐Vides, J. (2004). Successful lateral transfer requires codon usage compatibility between foreign genes and recipient genomes. Molecular Biology and Evolution, 21, 1884–1894. 10.1093/molbev/msh202 15240837

[ece34924-bib-0058] Meisel, H. , & Bockelmann, W. (1999). Bioactive peptides encrypted in milk proteins: Proteolytic activation and thropho‐functional properties. Antonie Van Leeuwenhoek, 76, 207–215. 10532380

[ece34924-bib-0059] Mira, A. , Ochman, H. , & Moran, N. A. (2001). Deletional bias and the evolution of bacterial genomes. Trends in Genetics, 17, 589–596. 11585665 10.1016/s0168-9525(01)02447-7

[ece34924-bib-0060] Moreno, M. , & Giralt, E. (2015). Three valuable peptides from bee and wasp venoms for therapeutic and biotechnological use: Melittin, apamin and mastoparan. Toxins, 7, 1126–1150. 10.3390/toxins7041126 25835385 PMC4417959

[ece34924-bib-0061] Mylonakis, E. , Podsiadlowski, L. , Muhammed, M. , & Vilcinskas, A. (2016). Diversity, evolution and medical applications of insect antimicrobial peptides. Philosophical Transactions of the Royal Society of London B: Biological Sciences, 371, 1695 10.1098/rstb.2015.0290 PMC4874388 27160593

[ece34924-bib-0062] Nabais, A. M. A. , & da Fonseca, M. M. R. (1995). The effect of solid medium composition on growth and sporulation of *Streptomyces clavuligerus*; spore viability during storage at +4°C. Biotechnology Techniques, 9, 361–364. 10.1007/BF00638871

[ece34924-bib-0063] Netolitzky, D. J. , Wu, X. , Jensen, S. E. , & Roy, K. L. (1995). Giant linear plasmids of beta‐lactam antibiotic producing *Streptomyces* . FEMS Microbiology Letters, 131, 27–34. 7557307 10.1016/0378-1097(95)00230-3

[ece34924-bib-0064] Perez, R. H. , Zendo, T. , & Sonomoto, K. (2014). Novel bacteriocins from lactic acid bacteria (LAB): Various structures and applications. Microbial Cell Factories, 13, S3 10.1186/1475-2859-13-S1-S3 25186038 PMC4155820

[ece34924-bib-0065] Petersen, T. N. , Brunak, S. , von Heijne, G. , & Nielsen, H. (2011). SignalP 4.0: Discriminating signal peptides from transmembrane regions. Nature Methods, 8, 785–786. 10.1038/nmeth.1701 21959131

[ece34924-bib-0066] Petre, B. , Major, I. , Rouhier, N. , & Duplessis, S. (2011). Genome‐wide analysis of eukaryote thaumatin‐like proteins (TLPs) with an emphasis on poplar. BMC Plant Biology, 11, 33 10.1186/1471-2229-11-33 21324123 PMC3048497

[ece34924-bib-0067] Porto, W. F. , Pires, A. S. , & Franco, O. L. (2017). Computational tools for exploring sequence databases as a resource for antimicrobial peptides. Biotechnology Advances, 35, 337–349. 10.1016/j.biotechadv.2017.02.001 28216008

[ece34924-bib-0068] Pruess, D. L. , & Kellett, M. (1983). Ro 22–5417, a new clavam antibiotic from *Streptomyces clavuligerus*. I. Discovery and biological activity. Journal of Antibiotics, 36, 208–212. 6833140 10.7164/antibiotics.36.208

[ece34924-bib-0069] Rawlings, N. D. , Barrett, A. J. , & Bateman, A. (2012). MEROPS: The database of proteolytic enzymes, their substrates and inhibitors. Nucleic Acids Research, 40, D343–350. 10.1093/nar/gkr987 22086950 PMC3245014

[ece34924-bib-0070] Reading, C. , & Cole, M. (1977). Clavulanic acid: A beta‐lactamase‐inhibiting beta‐lactam from *Streptomyces clavuligerus* . Antimicrobial Agents and Chemotherapy, 11, 852–857. 10.1128/AAC.11.5.852 879738 PMC352086

[ece34924-bib-0071] Richards, T. A. , Leonard, G. , Soanes, D. M. , & Talbot, N. J. (2011). Gene transfer into the fungi. Fungal Biology Reviews, 25, 98–110. 10.1016/j.fbr.2011.04.003

[ece34924-bib-0072] Schmitt, I. , & Lumbsch, H. T. (2009). Ancient horizontal gene transfer from bacteria enhances biosynthetic capabilities of fungi. PLoS ONE, 4, e4437 10.1371/journal.pone.0004437 19212443 PMC2636887

[ece34924-bib-0073] Sembiring, L. (2009). Molecular phylogenetic classification of *Streptomycetes* isolated from the rhizosphere of tropical legume (*Paraserianthes falcataria*) (L.) Nielsen. HAYATI Journal of Biosciences, 16, 100–108. 10.4308/hjb.16.3.100

[ece34924-bib-0074] Shetty, P. R. , Buddana, S. K. , Tatipamula, V. B. , Naga, Y. V. V. , & Ahmad, J. (2014). Production of polypeptide antibiotic from *Streptomyces parvulus* and its antibacterial activity. Brazilian Journal of Microbiology, 45, 303–312. 10.1590/S1517-83822014005000022 24948949 PMC4059315

[ece34924-bib-0075] Sieber, K. B. , Bromley, R. E. , & Dunning Hotopp, J. C. (2017). Lateral gene transfer between prokaryotes and eukaryotes. Experimental Cell Research, 358, 421–426. 10.1016/j.yexcr.2017.02.009 28189637 PMC5550378

[ece34924-bib-0076] Slot, J. C. , & Rokas, A. (2010). Multiple GAL pathway gene clusters evolved independently and by different mechanisms in fungi. Proceedings of the National Academy of Sciences USA, 107, 10136–10141. 10.1073/pnas.0914418107 PMC2890473 20479238

[ece34924-bib-0077] Song, J. Y. , Jeong, H. , Yu, D. S. , Fischbach, M. A. , Park, H.‐S. , Kim, J. J. , … Kim, J. F. (2010). Draft genome sequence of *Streptomyces clavuligerus* NRRL 3585, a producer of diverse secondary metabolites. Journal of Bacteriology, 192, 6317–6318. 10.1128/JB.00859-10 20889745 PMC2981214

[ece34924-bib-0078] Soucy, S. M. , Huang, J. , & Gogarten, J. P. (2015). Horizontal gene transfer: Building the web of life. Nature Reviews Genetics, 16, 472–482. 10.1038/nrg3962 26184597

[ece34924-bib-0079] Sun, J. , Xia, Y. , Li, D. , Du, Q. , & Liang, D. (2014). Relationship between peptide structure and antimicrobial activity as studied by de novo designed peptides. Biochimica Et Biophysica Acta (BBA) – Biomembranes, 1838, 2985–2993. 10.1016/j.bbamem.2014.08.018 25157672

[ece34924-bib-0080] Syvanen, M. (2012). Evolutionary implications of horizontal gene transfer. Annual Review of Genetics, 46, 341–358. 10.1146/annurev-genet-110711-155529 22934638

[ece34924-bib-0081] Tiburcio, R. A. , Costa, G. G. L. , Carazzolle, M. F. , Mondego, J. M. C. , Schuster, S. C. , Carlson, J. E. , … Pereira, G. A. G. (2010). Genes acquired by horizontal transfer are potentially involved in the evolution of phytopathogenicity in *Moniliophthora perniciosa* and *Moniliophthora roreri*, two of the major pathogens of cacao. Journal of Molecular Evolution, 70, 85–97. 10.1007/s00239-009-9311-9 20033398

[ece34924-bib-0082] Torrent, M. , Di Tommaso, P. , Pulido, D. , Nogués, M. V. , Notredame, C. , Boix, E. , & Andreu, D. (2012). AMPA: An automated web server for prediction of protein antimicrobial regions. Bioinformatics, 28, 130–131. 10.1093/bioinformatics/btr604 22053077

[ece34924-bib-0083] Tripathi, L. P. , & Sowdhamini, R. (2008). Genome‐wide survey of prokaryotic serine proteases: Analysis of distribution and domain architectures of five serine protease families in prokaryotes. BMC Genomics, 9, 549 10.1186/1471-2164-9-549 19019219 PMC2605481

[ece34924-bib-0084] Um, S. , Choi, T. J. , Kim, H. , Kim, B. Y. , Kim, S.‐H. , Lee, S. K. , … Oh, D.‐C. (2013). Ohmyungsamycins A and B: Cytotoxic and antimicrobial cyclic peptides produced by *Streptomyces* sp. from a volcanic island. Journal of Organic Chemistry, 78, 12321–12329. 10.1021/jo401974g 24266328

[ece34924-bib-0085] Van Arnam, E. B. , Currie, C. R. , & Clardy, J. (2018). Defense contracts: Molecular protection in insect‐microbe symbioses. Chemical Society Reviews, 47, 1638–1651. 10.1039/c7cs00340d 28745342

[ece34924-bib-0086] Vanhoye, D. , Bruston, F. , Nicolas, P. , & Amiche, M. (2003). Antimicrobial peptides from hylid and ranin frogs originated from a 150‐million‐year‐old ancestral precursor with a conserved signal peptide but a hypermutable antimicrobial domain. European Journal of Biochemistry, 270, 2068–2081. 12709067 10.1046/j.1432-1033.2003.03584.x

[ece34924-bib-0087] Veenstra, J. A. (2000). Mono‐ and dibasic proteolytic cleavage sites in insect neuroendocrine peptide precursors. Archives of Insect Biochemistry and Physiology, 43, 49–63. 10.1002/(SICI)1520-6327(200002)43:2>49:AID-ARCH1<3.0.CO;2-M 10644969

[ece34924-bib-0088] Veltri, D. , Kamath, U. , & Shehu, A. (2018). Deep learning improves antimicrobial peptide recognition. Bioinformatics, 34, 2740–2747. 10.1093/bioinformatics/bty179 29590297 PMC6084614

[ece34924-bib-0089] Venner, S. , Miele, V. , Terzian, C. , Biémont, C. , Daubin, V. , Feschotte, C. , & Pontier, D. (2017). Ecological networks to unravel the routes to horizontal transposon transfers. PLoS Biology, 15, e2001536 10.1371/journal.pbio.2001536 28199335 PMC5331948

[ece34924-bib-0090] Vernikos, G. S. , & Parkhill, J. (2006). Interpolated variable order motifs for identification of horizontally acquired DNA: Revisiting the *Salmonella* pathogenicity islands. Bioinformatics, 22, 2196–2203. 10.1093/bioinformatics/btl369 16837528

[ece34924-bib-0091] Waghu, F. H. , Barai, R. S. , Gurung, P. , & Idicula‐Thomas, S. (2016). CAMPR3: A database on sequences, structures and signatures of antimicrobial peptides. Nucleic Acids Research, 44, D1094–D1097. 10.1093/nar/gkv1051 26467475 PMC4702787

[ece34924-bib-0092] Wang, G. (2015). Improved methods for classification, prediction and design of antimicrobial peptides. Methods in Molecular Biology, 1268, 43–66. 10.1007/978-1-4939-2285-7_3 25555720 PMC4578715

[ece34924-bib-0093] Wang, G. (2017). Antimicrobial peptides: Discovery, design and novel therapeutic strategies (2nd ed.). Wallingford, UK: CABI.

[ece34924-bib-0094] Wang, G. , Li, X. , & Wang, Z. (2016). APD3: The antimicrobial peptide database as a tool for research and education. Nucleic Acids Research, 44, D1087–1093. 10.1093/nar/gkv1278 26602694 PMC4702905

[ece34924-bib-0095] Wang, X. , & Liu, X. (2016). Close ecological relationship among species facilitated horizontal transfer of retrotransposons. BMC Evolutionary Biology, 16, 201 10.1186/s12862-016-0767-0 27717306 PMC5055719

[ece34924-bib-0096] Wenzl, P. , Wong, L. , Kwang‐won, K. , & Jefferson, R. A. (2005). A functional screen identifies lateral transfer of beta‐glucuronidase (gus) from bacteria to fungi. Molecular Biology and Evolution, 22, 308–316. 10.1093/molbev/msi018 15483318

[ece34924-bib-0097] Woolfit, M. , Iturbe‐Ormaetxe, I. , McGraw, E. A. , & O’Neill, S. L. (2009). An ancient horizontal gene transfer between mosquito and the endosymbiotic bacterium Wolbachia pipientis. Molecular Biology and Evolution, 26, 367–374. 10.1093/molbev/msn253 18988686

[ece34924-bib-0098] Wu, B. , Novelli, J. , Jiang, D. , Dailey, H. A. , Landmann, F. , Ford, L. , … Slatko, B. E. (2013). Interdomain lateral gene transfer of an essential ferrochelatase gene in human parasitic nematodes. Proceedings of the National Academy of Sciences USA, 110, 7748–7753. 10.1073/pnas.1304049110 PMC3651471 23610429

[ece34924-bib-0099] Yi, H.‐Y. , Chowdhury, M. , Huang, Y.‐D. , & Yu, X.‐Q. (2014). Insect antimicrobial peptides and their applications. Applied Microbiology and Biotechnology, 98, 5807–5822. 10.1007/s00253-014-5792-6 24811407 PMC4083081

[ece34924-bib-0100] Zhang, L.‐J. , & Gallo, R. L. (2016). Antimicrobial peptides. Current Biology, 26, R14–19. 10.1016/j.cub.2015.11.017 26766224

[ece34924-bib-0101] Zhao, Q. , Garreau, I. , Sannier, F. , & Piot, J. M. (1997). Opioid peptides derived from hemoglobin: Hemorphins. Biopolymers, 43, 75–98. 10.1002/(SICI)1097-0282(1997)43:2<75:AID-BIP2>3.0.CO;2-X 9216245

[ece34924-bib-0102] Zhou, H. , Yang, Y. , Yang, X. , Li, W. , Xiong, Z. , Zhao, L. , … Ding, Z. (2014). A new cyclic tetrapeptide from an endophytic *Streptomyces* sp. YIM67005. Natural Product Research, 28, 318–323. 10.1080/14786419.2013.863198 24304298

[ece34924-bib-0103] Zhu, S. , & Gao, B. (2014). Nematode‐derived drosomycin‐type antifungal peptides provide evidence for plant‐to‐ecdysozoan horizontal transfer of a disease resistance gene. Nature Communications, 5, 3154 10.1038/ncomms4154 24434635

