# Supplementary material for: A putative antimicrobial peptide from Hymenoptera in the megaplasmid pSCL4 of Streptomyces clavuligerus ATCC 27064 reveals a singular case of horizontal gene transfer with potential applications
Source: Ecol Evol. 2019 Jan 30;9(5):2602–14. doi: 10.1002/ece3.4924 (PMC6406012; doi:10.1002/ece3.4924)
Supplement: Supplementary file 1 [file ECE3-9-2602-s001.docx]

Supplementary Table 1. Software and sequences used to calculate the sensibility and specificity of AMP predictors

| AMP^a^ | Software^b^ | CAMP3  (SVM, RF, ANN and DA) | AMPA  (predilection scale) | ClassAMP  (SVM and RF) | AntiBP  (SVM, ANN and QM) | MLAMP  (ML-SMOTE) | ADP3  (similarity) | AMP Scanner Vr.2  (DNN) |
| --- | --- | --- | --- | --- | --- | --- | --- | --- |
| Positive controls | | | | | | | | |
| Cecropin2 (AP00135) | | * | * | * | * | * | * | * |
| Melittin (AP00146) | | * |  | * | * | * | * | * |
| BM Moricin (AP00147) | | * | * | * | * | * | * | * |
| Mastoparan B (AP00200) | | * |  | * | * | * | * | * |
| Thanatin (AP00102) | | * |  | * | * | * | * | * |
| Alo-3 (AP00813) | | * |  | * | * |  | * | * |
| Tachystatin B1 (AP01005) | | * | * | * | * | * | * | * |
| Psalmopeotoxin I (AP02553) | | * | * | * | * |  | * | * |
| Drosomycin (AP00672) | | * |  | * | * | * | * | * |
| Phormicin (AP00216) | | * | * | * | * | * | * | * |
| Heliomicin (AP00031) | | * | * | + | * | * | * | * |
| Spiderine OtTx1a (AP02558) | | * | * | * | * |  | * | * |
| Pyrrhocoricin (AP00170) | | + |  | * | * | * | * | * |
| Drosocin (AP00172) | | * |  | * | + |  | * | * |
| Drosophila diptericin (AP01565) | | + |  | * | * |  | * | * |
| SsALF (AP02259) | | * | * | * | * |  | * | * |
| Alloferon 2 (AP00024) | |  |  | * | * | * | * |  |
| Ponericin G2 (AP00377) | | * | * | * | * | * | * | * |
| Pilosulin 3 (AP00891) | | * | * | * | * | * | * | * |
| Ponericin G5 (AP00380) | | * | * | * | * | * | * | * |
| Total AMPs predicted | | 19 | 11 | 20 | 20 | 14 | 20 | 19 |
| Negative controls | | | | | | | | |
| General transcription factor IIA subunit 2 (P52656) | | + | * | * | * |  | * |  |
| Replication initiator 1-like  (XP_023011967) | | + | * | * | * |  | * | * |
| Single-stranded DNA-binding protein (XP_012282296) | | * | * | * | * |  | * |  |
| Enhancer of yellow 2 transcription factor  (Q9VYX1) | | + |  | * | * |  |  |  |
| Rad51D (ALC40653) | | + | * | * | * |  |  | * |
| Mediator of RNA polymerase II transcription subunit 21  (Q9W5P1) | | + |  | * | * |  |  |  |
| Replication protein A 14 kDa subunit (KOC62485) | | + |  | * | * |  | * |  |
| Mediator of RNA polymerase II transcription subunit 10 (Q16G71) | | + |  | * | * |  | * |  |
| Transcription initiation factor IIA subunit 2 (XP_011137704) | | + | * | * | * |  | * |  |
| Transcription factor IIIB 90 kDa subunit (KYQ49479) | | * |  | * | * |  | * |  |
| TATA binding protein asssociated factor 24kDa subunit (CAB55760) | | + |  | + | * |  |  |  |
| Zinc finger protein 70 (ABF18021) | | + | * | * | * |  | * | * |
| Transcription elongation factor SPT4 (XP_001652051) | | + | * | * | * |  | * | * |
| Transcription factor AP-2-epsilon (XP_021207020) | | * | * | * | * |  | * | * |
| Replication protein A3, isoform A  (AAF48070) | | + |  | * | * |  | * |  |
| Replication factor A subunit (JAQ02219) | | * |  | * | * |  | * |  |
| DNA replication factor Cdt1 (XP_001870780) | | * | * | * | * |  | * | * |
| DNA replication complex GINS protein PSF2 (PNF43610) | | + | * | * | * |  | * |  |
| Replication protein A 14 kDa subunit B (XP_023291404) | | + |  | * | * |  |  |  |
| DNA replication inhibitor plutonium (XP_023306194) | | * | * | * | * |  | * | * |
| **Total AMPs predicted** | | **20** | **11** | **20** | **20** | **0** | **15** | **7** |
| **Specificity (%)** | | **0** | **45** | **0** | **0** | **100** | **25** | **35** |
| **Sensitivity (%)** | | **95** | **55** | **100** | **100** | **70** | **100** | **95** |

*Predicted by all methods

^+^ Predicted by one method

^a^ The positive control codes come from the APD and the negative control codes come from the NCBI

^b^ Algorithms used by each software. SVM - Support vector machine, RF – Random forest, ANN - Artificial neural network, DA Discriminant analysis , QM - Quantitative Matrices, ML-SMOTE - Multilabel Synthetic Minority Over-sampling Technique, DNN - Deep neural network
